# Effects of an exercise program with augmented reality on functional fitness and physical activity of community-dwelling older adults

**DOI:** 10.3389/fspor.2024.1447866

**Published:** 2025-01-07

**Authors:** Soraia Ferreira, José Marmeleira, Jesus Del Pozo Cruz, Nilton Leite, Alexandre Bernardino, Ana Moradell, Armando Raimundo

**Affiliations:** ^1^ Department of Sport and Health, School of Health and Human Development, University of Evora, Évora, Portugal; ^2^ Comprehensive Health Research Center, New University of Lisbon, Lisboa, Portugal; ^3^ Department of Physical Education and Sports, University of Seville, Seville, Spain; ^4^ Epidemiology of Physical Activity and Fitness Across Lifespan Research Group (EPAFit), University of Seville, Seville, Spain; ^5^ Laboratory for Robotics and Engineering Systems (LARSyS), Instituto Superior Técnico (ISR), Lisboa, Portugal; ^6^ Growth, Exercise, Nutrition and Development (EXER-GENUD) Research Group, Universidad de Zaragoza, Zaragoza, Spain; ^7^ Instituto de investigación Sanitaria de Aragón (IIS Aragón), Zaragoza, Spain; ^8^ Instituto Agroalimentario de Aragón-IA2 (Universidad de Zaragoza-CITA), Zaragoza, Spain; ^9^ Department of Animal Production and Food Sciences, Faculty of Health and Sport Sciences, University of Zaragoza, Zaragoza, Spain

**Keywords:** physical function, physical activity, older adults, augmented reality, multimodal training

## Abstract

**Background:**

This study aims to investigate the effects of a multimodal program using augmented reality on the functional fitness and physical activity of older adults living in the community.

**Method:**

Seventy-eight older adults living in the community participated in this study. Participants were divided into three groups: a control group that maintained their usual activities, and two experimental groups, one with multimodal training (EG1) and the other with multimodal training combined with augmented reality (EG2). Participants were assessed at baseline and post-intervention, after 12 weeks. Functional fitness was assessed using the Rikli and Jones Senior Fitness Test, handgrip strength, the functional reach test, and the Fullerton Advanced Balance Scale. Physical activity was measured using accelerometry.

**Results:**

In EG1, lower limb flexibility, agility, cardiorespiratory fitness, and balance improved significantly between baseline and the 12-week outcome (*p* ≤ 0.001 for all). In EG2, improvements were observed in upper and lower limb strength, lower limb flexibility, agility, cardiorespiratory fitness, handgrip strength, and balance (*p* < 0.05 for all). Sedentary behavior increased in EG1 after the intervention. The clinical effect sizes of the interventions were large for balance (ES = 1.19) in EG1 and for upper limb strength (ES = 1.24) in EG2, and medium for cardiorespiratory fitness (ES = 0.74), agility (ES = 0.50), and lower limb flexibility (ES = 0.65) in EG1, and lower limb strength (ES = 0.61) and cardiorespiratory fitness (ES = 0.79) in EG2.

**Conclusion:**

Both intervention programs led to improvements in several functional domains. However, the multimodal training combined with augmented reality program showed improvements across more domains, resulting in greater changes. Physical activity did not show significant improvements.

## 1 Introduction

In the context of aging, there is a tendency for functional fitness and physical activity to decrease (1). Functional fitness (FF) plays a key role in the daily life of the population, as it is associated with the performance of daily living tasks and the maintenance of independence (2, 3). The concept of FF is associated with cardiorespiratory fitness, balance, strength, agility, flexibility, and body composition (4). On the other hand, physical activity allows people to gain several benefits, not only physically, but also cognitively and psychologically (5–7).

Over the past few years, various studies have been conducted to investigate the effects of different exercise programs on functional fitness and physical activity in the elderly. These exercise programs have included aerobic, multimodal, balance, and aquatic training (8, 9). Recently, there has been a growing interest in combining new technologies with physical activity, leading to the development of exercise programs utilizing virtual reality (VR) and exergames (10, 11). Exergames have been shown to motivate participants to be physically active by promoting enjoyment and fun during exercise. However, most exergames are not specifically designed to increase recommended levels of physical activity (12).

Virtual reality (VR) training has demonstrated improvements in functional fitness, balance, and cognitive abilities in older adults (10, 13, 14), but it also presents some challenges, such as issues with face-to-face communication, simulation sickness, headaches, and safety concerns (15, 16). In addition, VR can be an alternative to the use of exergames, but not a substitute (17).

In parallel with virtual reality, augmented reality (AR) has also been explored in some contexts, especially in balance training (18). One of the major benefits of AR is that it provides users with virtual experiences in the real world, allowing them to have new and safer experiences without exposing older people to dangerous environments (19).

Recently, AR has been increasingly associated with physical activity, and some exercise programs have already been developed using this technology. The use of AR for older adults can offer various benefits, promoting a healthy lifestyle by enhancing the performance of daily tasks and supporting rehabilitation (20). Gonçalves et al. (21) observed that participants spent more time in moderate to vigorous intensity physical activity when exercising with AR than traditional fitness training. However, research linking AR and physical activity is limited (19).

Recent studies have reported high self-efficacy in exercises performed with AR, and improvements in lower limb strength, cardiorespiratory fitness, mobility, and maximal inspiratory pressure (22, 23). To our knowledge, there has been only one study examining the effects of AR on functional fitness in older adults, which was conducted in a real-world setting. Therefore, this study emerged from the need to better understand the effects of interventions that combine exercise and new technologies on physical fitness in older adults. Thus, this study aims to investigate the effects of an exercise intervention using multimodal exercise with augmented reality and multimodal exercise alone on the functional fitness and physical activity behavior of older adults living in the community.

## 2 Method

### 2.1 Study design and participants

Seventy-eight community-dwelling older adults participated in this study. The research project was promoted through posters and flyers distributed throughout the region. Individuals interested in participating visited the University gymnasium to register. Participants were eligible for the study if they were 60 years of age or older and had motor independence (unassisted). Exclusion criteria included the presence of a pacemaker and cognitive impairment. The Mini-Mental State Examination (MMSE) was used to assess cognitive impairment. This test evaluates six cognitive domains, and we applied the cutoff scores from the Portuguese version of the MMSE: ≤27 for individuals with more than 11 years of education, ≤22 for those with 1–11 years of education, and ≤15 for illiterate individuals (24). Table 1 contains the characteristics of the participants.

**Table 1:** Descriptive analysis of all participants, by groups. Results are expressed as mean ± SD.

	CG	EG2	EG1	*p*
Age (years)	71.50 (3.9)	72.14 (6.1)	73.52 (7.4)	0.530*
Weight (kg)	76.38 (12.1)	76.04 (10.5)	69.34 (13.3)	0.108*
Height (cm)	158.84 (8.4)	160.72 (8.1)	155.32 (9.8)	0.139*
BMI (kg/m^2^)	30.32 (4.6)	29.45 (3.5)	28.65 (4.1)	0.417*
Education (years)	6.23 (4.7)	7.0 (3.6)	9.19 (5.0)	0.091*
SBP (mm Hg)	142.33 (15.7)	130.43 (19.3)	146.15 (17.3)	0.015*^,^**
DBP (mm Hg)	81.24 (12.1)	81.76 (16.2)	81.15 (10.6)	0.987*
Waist circumference (cm)	105.37 (18.8)	100.73 (11.2)	95.91 (11.2)	0.104*
Hip circumference (cm)	104.60 (13.4)	103.15 (6.6)	101.40 (7.3)	0.560*

CG, control group; EG2, experimental group with augmented reality; EG1, experimental group with multimodal training; BMI, body mass index; SBP, systolic blood pressure; DBP, diastolic blood pressure.

* Anova test *p*-value.

** *p* > 0.05.

**Figure 1:**
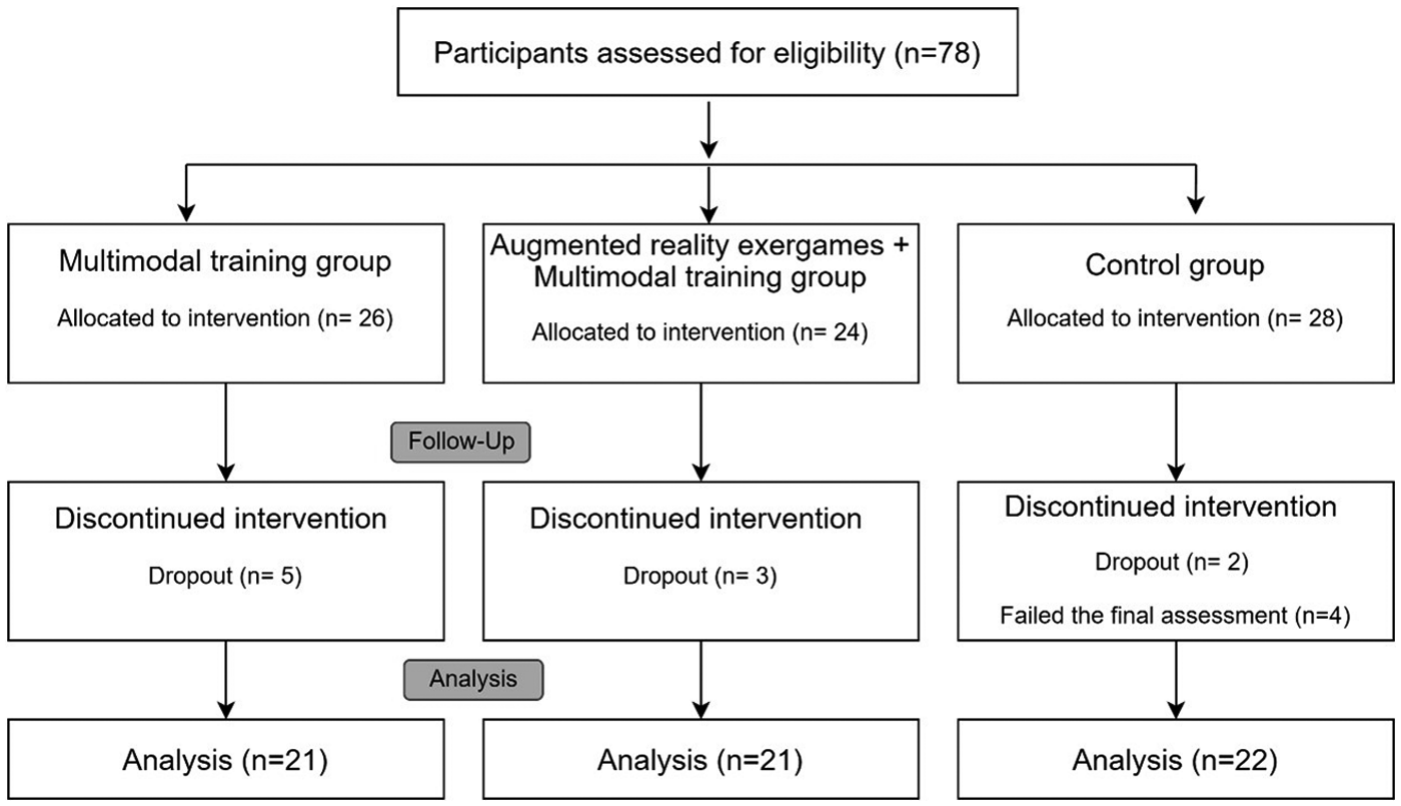
Flow diagram of participant's recruitment.

Participants eligible for the study were divided into groups based on their availability, as randomizing the participants was not feasible (Figure 1). There were three different groups: a control group (CG), an experimental group with multimodal training (EG1) and an experimental group with multimodal AR training (EG2). Twenty-eight participants participated in the CG, 26 in EG1 and 24 in EG2. All participants were informed of the study's aims and gave informed consent before participation. The study was approved by the University of Évora's Ethics Committee (GD/21849/2017) and was conducted in accordance with the Declaration of Helsinki. The protocol was registered in ClinicalTrials.gov (NCT05727748).

### 2.2 Outcomes

Participants underwent two assessments, one at the beginning and one at the end of the program. The participants underwent training on some of the tests that were deemed necessary immediately before the evaluation testing session. The assessments were conducted by an exercise physiologist and lasted approximately one and a half hours.

Physical fitness was assessed by the Senior Fitness Test, which assessed lower and upper limb flexibility, cardiorespiratory fitness, upper and lower limb strength, and agility. These variables were respectively assessed by (1) chair sit and reach (cm): the participant sat on the edge of a chair with one leg extended straight out in front. They were instructed to reach forward with their hands toward the toes of the extended leg while keeping the opposite leg bent and the foot flat on the floor. The distance between the fingertips and the toes was measured. A positive value was recorded if the fingertips passed the toes, and a negative value if they did not reach the toes; (2) back scratch (cm): the participant reached one hand over their shoulder and the other hand up their back, attempting to make the fingers meet. The distance between the fingertips was measured, with a positive score indicating that the fingers overlapped and a negative score indicating that they did not meet; (3) 6 min walk (m): the participant was instructed to walk as far as possible, without running, within 6 min in a predefined area. The total distance covered was measured; (4) 30-s chair stand (repetitions): the participant sat in a chair with arms crossed over the chest and was instructed to stand up fully and sit down again as many times as possible within 30 s. The number of complete stands was counted; (5) arm curl (repetitions): while seated, the participant held a weight (5 pounds for women and 8 pounds for men) in their dominant hand and performed as many bicep curls as possible within 30 s; and (6) timed up and go (s): the participant began seated in a chair. On a signal, they stood up, walked 2.44 m, turned around, walked back to the chair, and sat down as quickly as possible. The time taken to complete the entire sequence was recorded in seconds (25).

Here is the corrected and refined version of the text:

Balance was assessed using the Functional Reach Test (FRT) and the Fullerton Scale. The FRT is performed while the participant stands upright with a stable base. The task requires the participant to reach forward with an outstretched arm without moving their feet. The difference between the starting and ending points (in cm) is measured. Each participant performs the exercise three times, and the average is used for statistical analysis (26). The Fullerton Scale was developed for independent older adults and assesses both static and dynamic balance. It consists of 10 activities: standing with eyes closed and feet together, turning in a circle, walking up and down a step, tandem walking, one-legged balance, standing with eyes closed on a foam surface, jumping a horizontal distance, walking while turning the head, and regaining balance after an unexpected loss. Each activity is scored from 0 to 4, with 0 indicating the inability to perform the task and 4 representing the best possible performance (27).

The handgrip dynamometer (HGD) was used to measure hand and forearm muscle strength (in kilograms) (Baseline Smedley, model 12-0286, White Plains, NY, USA). The test was performed seated with the elbow flexed at 90° and the wrist grip individually adjusted for each participant. Participants were instructed to reach their maximum strength in 3 trials. The test was performed for both the dominant (HGDH) and nondominant hand (HGNDH). The average of the 3 trials for each hand was used for statistical analysis (28).

Physical activity was measured with an accelerometer (ActiGraph wGT3X- BT; ActiGraph, LLC, Pensacola, Florida). Previous research has shown that accelerometer measurement is valid for quantifying physical activity in adults (29). Participants were asked to wear the accelerometer 24 h a day for 7 consecutive days. The accelerometer was removed for water activities and bathing. The device was worn at the level of the right hip. Activation and downloading of accelerometer data were performed via Actilife software. The results of participants who used the accelerometer on at least 3 valid days, including one day during the weekend, were considered. The variables measured with the accelerometer were sleep duration, sedentary time, and physical activity (light, moderate, vigorous, moderate-vigorous, and total). The criteria used to define PA were: sedentary: <100 counts per minute; light: 100–2,019 counts per minute; moderate: 2,020–5,998 counts per minute; vigorous: >5,999 counts per minute (30).

### 2.3 Intervention program

The intervention program lasted 12 weeks, with initial and final assessments conducted for all participants. The control group continued their daily activities without any specific physical activity intervention. The experimental groups participated in 60-min exercise sessions three times per week. Each session in both experimental groups began with a 6-min warm-up and joint mobilization and ended with a 6-min cool-down period.

In EG1, sessions were structured around four stations: a strength station, a cardiorespiratory fitness station, a dual-task reaction time station, and a dual-task agility and coordination station. Each participant spent 12 min at each station, completing all four stations during each session At the strength station, exercises were performed using body weight, dumbbells, and resistance bands. Initially, participants began with bodyweight exercises to learn proper technique, and gradually progressed to using dumbbells and resistance bands. The difficulty was progressively increased by raising the number of repetitions, the weight of the dumbbells, and the resistance of the bands, with the progression tailored to the individual characteristics of each participant. The cardiorespiratory fitness station involved walking exercises, where obstacles were added to the course over time, and participants carried an external load, such as 0.5 kg dumbbells in each hand. Stairs were also introduced, and the walking pace was gradually increased. The dual-task reaction time station required participants to complete a circuit that involved performing a motor task while simultaneously responding as quickly as possible to a stimulus. Meanwhile, at the dual-task agility and coordination station, exercises involved using a ball over a 20-m course while engaging in a cognitive task. For example, participants might throw a ball in the air and tap the ground twice while counting backward from 50 by twos, or bounce the ball once with the right hand, then three times with the left hand, while adding numbers in increments of three. The transition time between stations served as rest, and participants were also allowed to rest whenever needed. The training sessions and their respective stations were individually adapted to suit the specific characteristics of each participant.

EG2 sessions consisted of six stations focusing on training strength, cardiorespiratory fitness, dual-task reaction time, and dual-task agility and coordination. Participants in this group stayed at each station for 8 min. Four of the stations were the same as those used in EG1, with the addition of two stations incorporating augmented reality (AR) technology. The same physical and cognitive variables were targeted at these AR stations, but with the enhancement of AR features.

**Figure 2:**
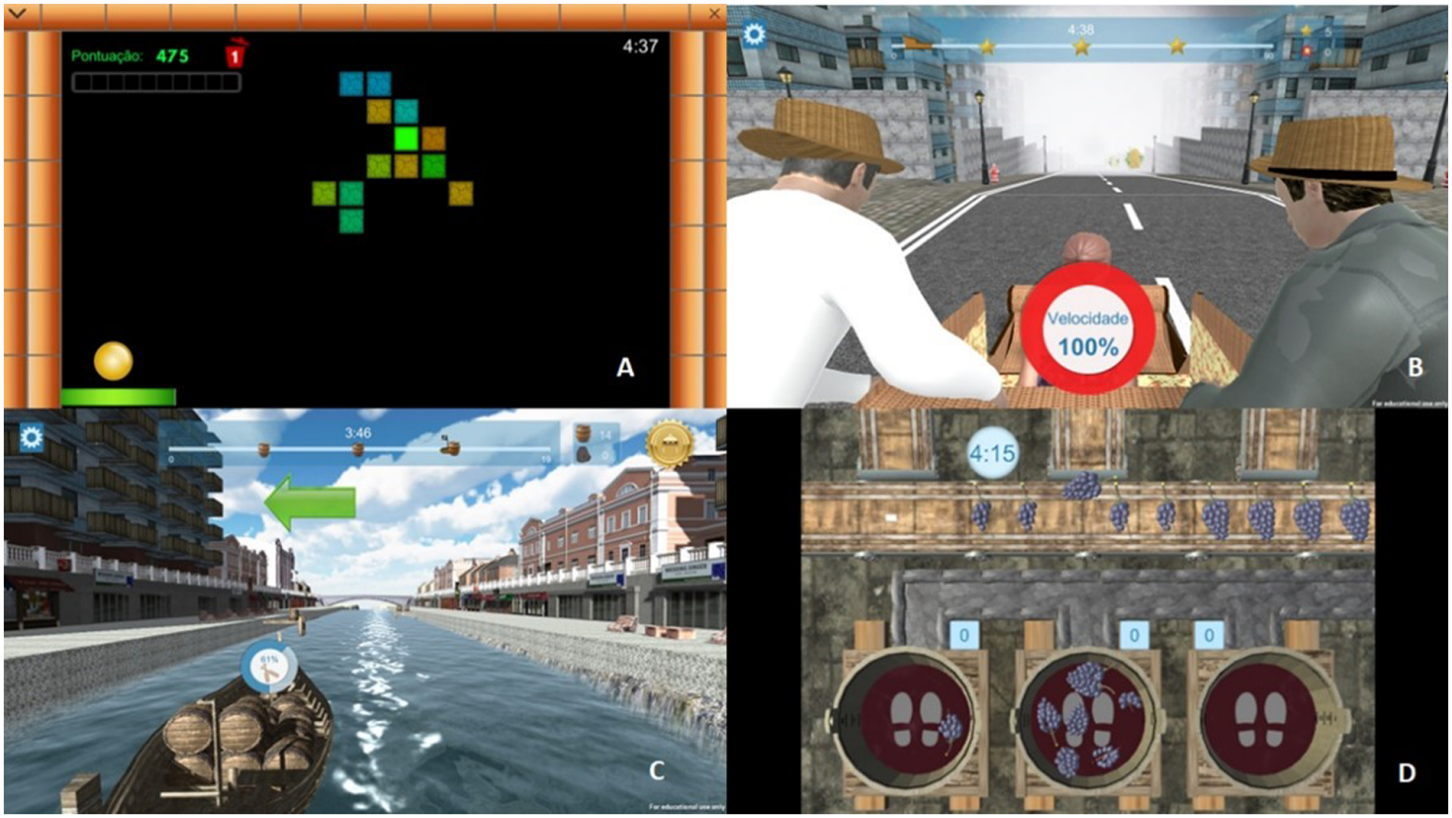
Different games on the portable exergame platform for elderly. **(A)** Exerpong; **(B)** toboggan games; **(C)** rabelos; **(D)** grape stomping.

At one of the AR stations, the training was conducted using the Portable Exergame Platform for Elderly (PEPE) (referred to as AR Station 1), while the other station involved activities projected on a wall (referred to as AR Station 2). The activities at both AR stations were specifically designed for the elderly population. PEPE is a platform comprising five exergames, and in this study, four of these games were utilized: Exerpong, Grape Stomping, Rabelos, and Toboggan Games (Figure 2). These games targeted cardiorespiratory fitness, coordination, strength, agility, and reaction time, with most activities performed as dual tasks. The technology implemented in PEPE is detailed in the study by Gonçalves (21), and the methodology applied was consistent with that used in a previously published study by Ferreira et al. (31, 32). The exergames were designed to simultaneously train physical and cognitive variables, with a different exergame being featured in each session. At AR Station 2, there were four distinct activities, including tasks such as touching a circle as quickly as possible to make it disappear and marching on the spot while responding to a predefined stimulus with the palm of the hand.

At the end of the training sessions, the Borg CR10 scale was used to monitor training intensity, and the Nasa TLX was used to control the mental and physical effort perceived by the participants, considering the entire exercise session.

### 2.4 Data analysis

The sample size was determined utilizing G*Power, an analytical software, with the aid of the RStudio platform, incorporating the pwr library. The calculations were based on an effect size of 0.573, an α level of 0.05, and a statistical power of 0.8. Consequently, a recommended sample size of 11 older adults was proposed for each experimental group. The effect size used was based on results of meta-analyses conducted on the effects of programs with augmented reality or exergames on older adults. Normality analysis was calculated using the Shapiro-Wilk test. Parametric tests were used for the normally distributed variables, and nonparametric tests were used for the nonnormally distributed variables. For the normally distributed variables, the ANOVA was used for comparison between groups, and paired sample *t*-test was used to compare the preintervention and postintervention periods within the same group. For nonparametric variables, the independent-samples *t*-test was used to compare groups, and the Wilcoxon test was used to compare the moment before and after the intervention within the same group. For comparison between groups, the delta value (Δ: moment1-moment0) was used. Their proportional change value (Δ%=[(moment1−moment0)/moment0]×100) was calculated for all variables between the preintervention and postintervention periods.

The effect size (Cohen's d) and 95% CI were calculated to determine the magnitude of the treatment effect and the clinical significance of the interventions. Cohen's cutoff values were used, with 0.20, 0.50, and 0.80 indicating a small, medium, and large effect, respectively (33).

Descriptive analysis was used for all variables, considering the mean and standard deviation. Analyses were performed using PASW Statistical for Windows statistical software (version 22.0; IBM SPSS Inc). For all statistical tests, significance was set at *p* < 0.05.

## 3 Results

In this study, 78 participants were assigned to the group that was most convenient for them. Within the multimodal training group, 5 participants withdrew from the study, while 21 successfully completed the intervention. Similarly, 21 participants completed the intervention within the multimodal training group with augmented reality (AR), while 22 were part of the control group. Among the initial 28 participants in the control group, 5 dropped out, and 2 did not finish the final assessments. The participants who dropped out did so because they had to attend to family matters and no longer had time to participate in the exercise sessions. Descriptive characteristics such as weight, height, age, education level, body mass index, and resting heart rate did not differ significantly among the three groups.

A total of 36 exercise sessions were conducted, and participants were required to attend at least 80% of these sessions to be included in the study. All participants who began the intervention met this attendance requirement and completed both the initial and final assessments. Overall, the exercise sessions had high attendance rates. In the EG2 group, 10 participants attended over 90% of the sessions, while 11 attended between 80% and 90%. In the EG1 group, 17 participants attended at least 90% of the sessions, and 4 were present for more than 80%.

Initially, the physical fitness of all participants was assessed to determine their baseline condition before starting the program. The results were then compared with the reference values proposed by Mendes et al. (2014), based on the age and sex of each participant. Across all groups, more than 70% of participants exceeded the recommended values for upper and lower limb strength, agility, and cardiorespiratory fitness. In terms of flexibility, 50% of participants in the control group (CG) had values above the reference, while in the multimodal training groups (EG1 and EG2), 60% of participants exceeded the recommended levels for their age and sex.

Table 2 shows the results of the effects of the different intervention programs on functional fitness. After the 12-week intervention, CG showed worse outcomes and more time on the TUG (Δ% = 4.78%) and increased back scratching distance (Δ% = 18.73%) compared to the pre-intervention and post-intervention periods. EG2 showed better results in upper limb strength (Δ% = 21.48%) and lower limb strength (Δ% = 8.77%) after the 12-week of intervention. In the test of nondominant handgrip, EG2 showed improvement in performance after the 12 weeks (Δ% = 9.13%). EG2 and EG1 showed better results in the chair sit-and-reach test (EG2, Δ% = −77.12%; EG1; Δ% = −82.18%), in the TUG (EG2, Δ% = −6.60%; EG1, Δ% = −7. 08%), in the cardiorespiratory fitness test (EG1, Δ% = 9.62%; EG2, Δ% = 5.94%), and in the balance test (EG1, Δ% = 13.79%; EG2, Δ% = 15.01%), with improved performance in all tests. Finally, on FRT, EG2 (Δ% = 21.88%) showed significant improvements and increased range.

**Table 2:** Impact of the augmented reality and multimodal exercise programs on fitness function.

	Baseline mean (SD)	Post-intervention mean (SD)	*p*-value	Effect size Cohen's d (95% CI)	Δ%
30-s chair stand (rep)					
EG1	13.48 (3.1)	14.33 (3.6)	0.176**	0.26 (−0.36, 0.85)	6.31%
EG2	12.43 (1.6)	13.52 (1.9)	0.030**^,^***	0.61 (−0.03, 1.19)	8.77%
CG	13.55 (3.5)	12.68 (3.6)	0.098**	−0.25 (−0.87, 0.38)	−6.42%
Arm curl (rep)					
EG1	15.70 (2.8)	16.52 (2.9)	0.120**	0.29 (−0.32, 0.94)	5.22%
EG2	14.90 (2.2)	18.10 (2.9)	0.001*^,^***	1.24 (0.65, 1.75)	21.48%
CG	17.73 (3.6)	16.09 (4.5)	0.096**	−0.40 (−0.99, 0.21)	−9.25%
Back scratch (cm)					
EG1	−13.59 (9.01)	−12.09 (10.5)	1.00**	0.15 (−0.49, 0.76)	−11.04%
EG2	−9.57 (10.8)	−9.39 (11.4)	0.394**	0.02 (−0.62, 0.65)	−1.88%
CG	−15.38 (10.6)	−18.26 (11.4)	0.028**^,^***	−0.26 (−0.84, 0.37)	18.73%
Chair sit-and-reach (cm)					
EG1	−9.37 (11.9)	−1.67 (11.5)	0.001*^,^***	0.65 (0.02, 1.3)	−82.18%
EG2	−4.59 (12.6)	−1.05 (13.8)	0.042**^,^***	0.27 (−0.37, 0.9)	−77.12%
CG	−6.45 (14.2)	−6.05 (15.9)	0.776**	0.03 (−0.59, 0.63)	−6.20%
8 ft up-and-go (s)					
EG1	6.07 (0.86)	5.64 (0.86)	0.011**^,^***	−0.50 (−1.15, 0.176)	−7.08%
EG2	6.36 (1.0)	5.94 (1.0)	0.006*^,^***	−0.41 (−0.99, 0.25)	−6.60%
CG	6.28 (1.3)	6.58 (1.3)	0.033**^,^***	0.23 (−0.38, 0.84)	4.78%
6-minute walk (m)					
EG1	472.26 (27.5)	500.33 (45.3)	0.001**^,^***	0.74 (0.06, 1.43)	5.94%
EG2	440.55 (52.9)	482.93 (54.9)	0.007**^,^***	0.79 (0.145, 1.39)	9.62%
CG	422.59 (161.7)	454.46 (131.6)	0.249*	−0.03 (−0.623, 0.572)	7.54%
FRT (cm)					
EG1	28.56 (5.3)	34.81 (5.2)	<0.001**^,^***	1.19 (0.43, 1.85)	21.88%
EG2	31.44 (5.9)	32.49 (5.8)	0.501**	0.18 (−0.46, 0.81)	3.34%
CG	30.61 (6.8)	30.16 (4.9)	0.733**	−0.07 (−0.71, 0.52)	−1.47%
HGDH (Kg)					
EG1	25.6 (10.1)	24.00 (8.6)	0.185*	0.15 (−0.49, 0.77)	−6.25%
EG2	27.04 (9.3)	28.36 (7.3)	0.256**	0.16 (−0.46, 0.81)	4.88%
CG	25.38 (6.7)	25.56 (6.2)	0.778**	0.03 (−0.58, 0.63)	0.71%
HGNDH (Kg)					
EG1	23.32 (7.9)	22.61 (7.4)	0.357*	−0.09 (−0.71, 0.53)	−3.04%
EG2	23.55 (7.1)	25.70 (7.3)	0.011**^,^***	0.29 (−0.34, 0.91)	9.13%
CG	23.22 (5.9)	23.85 (5.8)	0.581**	0.11 (−0.49, 0.72)	2.71%
Balance (points)					
EG1	30.38 (4.9)	34.57 (3.9)	<0.001**^,^***	1.19 (0.53, 1.75)	13.79%
EG2	30.52 (4.7)	35.10 (2.7)	<0.001*^,^***	−0.24 (−0.84, 0.36)	15.01%
CG	29.82 (4.7)	28.64 (5.1)	0.185**	0.94 (0.30, 1.63)	−3.96%

EG2, experimental group with augmented reality and multimodal training; EG1, experimental group with multimodal training; FRT, functional reach test; HGDH, hand grip dominant hand; HGNDH, hand grip non-dominant hand.

* Wilcoxon test *p*-value.

** Paired-samples *t*-test *p*-value.

*** *p* ≤ 0.05.

When comparing the 3 groups regarding functional fitness, there are significant differences in lower (*p* = 0.020) and upper limb strength (*p* < 0.001), chair sit-and-reach (*p* = 0.035), 8 ft up-and-go (*p* < 0.001), functional reach test (*p* = 0.003), HGNDH (*p* = 0.020), and balance (*p* < 0.001). CG and EG2 showed significant differences in chair stance (*p* = 0.032), arm flexion (*p* = 0.001), and TUG (*p* = 0.002), with EG2 showing better results than CG. The values in TUG (*p* < 0.001), CSR (*p* = 0.013), FRT (*p* = 0.003) and balance (*p* < 0.001) showed significant differences when comparing CG and EG1, with the experimental group showing better results. When comparing the two experimental groups, EG2 showed better scores on the HGNDH (*p* = 0.016) than EG1, and EG1 better scores than EG2 on the Functional Reach Test (*p* = 0.031).

Table 3 presents the results related to physical activity, highlighting significant differences between groups in sedentary behavior and sleep. Changes in physical activity variables, as shown in Table 3, were observed after the 12-week intervention using accelerometry. Notably, only EG1 exhibited significant differences between the beginning and end of the intervention in the sedentary behavior and sleep variables. However, after 12 weeks, sedentary behavior increased (Δ% = 7.02%) and sleep duration decreased (Δ% = −9.02%). When comparing the 3 groups, there are significant differences in sleep (*p* = 0.05) and sedentary behavior (*p* = 0.04). GE1 showed better results as they had less sedentary behavior (*p* = 0.03) and more sleep time (*p* = 0.05) than GE2.

**Table 3:** Impact of the augmented reality and multimodal exercise programs on physical activity.

	Baseline mean (SD)	Post-intervention mean (SD)	*p*-value	Effect size Cohen's d (95% CI)	Δ%
Sedentary behavior (min/day)					
EG1	806.75 (119.2)	863.36 (113.0)	0.024**^,^***	0.49 (−0.18, 1.1)	7.02
EG2	843.2 (124.3)	802.67 (145.68)	0.178**	−0.29 (−0.95, 0.34)	−4.81
CG	847.02 (117.3)	865.11 (117.4)	0.678**	0.15 (−0.45, 0.81)	2.14
Total PA (min/day)					
EG1	104.75 (43.2)	88.55 (39.3)	0.079**	−0.39 (−1.02, 0.24)	−15.47
EG2	121.02 (67.4)	138.1 (102.8)	0.566**	0.19 (−0.41, 0.75)	14.11
CG	82.39 (44.3)	76.71 (43.1)	0.236**	−0.13 (−0.71, 0.49)	−6.89
Light PA (min/day)					
EG1	85.31 (37.2)	73.83 (33.4)	0.159**	−0.33 (−0.95, 0.30)	−13.46
EG2	98.36 (51.5)	111.99 (99.9)	0.627*	0.17 (−0.44, 0.77)	13.86
CG	68.41 (34.3)	68.81 (35.7)	0.322**	0.01 (−0.61, 0.61)	0.58
Moderate PA (min/day)					
EG1	19.43 (14.5)	14.72 (12.5)	0.058*	−0.35 (−0.89, 0.35)	−24.24
EG2	28.67 (30.6)	26.07 (28.4)	0.126*	−0.09 (−0.73, 0.55)	−9.07
CG	13.96 (14.8)	11.56 (15.1)	0.876*	−0.16 (−0.76, 0.51)	−17.19
MVPA (min/day)					
EG1	19.44 (14.5)	14.72 (12.5)	0.058*	−0.35 (−0.89, 0.35)	−24.28
EG2	27.34 (30.5)	26.12 (28.5)	0.274*	−0.04 (−0.66, 0.59)	−4.46
CG	13.97 (14.8)	11.04 (14.9)	0.615*	−0.19 (−0.82, 0.44)	−20.97
Vigorous PA (min/day)					
EG1	0.001 (0.0)	0.001 (0.0)	1.00*		0.00
EG2	0.04 (0.1)	0.06 (0.2)	1.00*	0.11 (−0.61, 0.66)	50.00
CG	0.01 (0.0)	0.01 (0.0)	0.596*	−0.09 (−0.65, 0.57)	0.00
Sleep time (min/day)					
EG1	478.61 (105.7)	435.42 (102.3)	0.030**^,^***	−0.42 (−1.03, 0.23)	−9.02
EG2	425.93 (122.4)	454.59 (132.3)	0.126**	0.23 (−0.43, 0.87)	6.73
CG	455.71 (99.7)	446.78 (103.2)	0.821**	−0.09 (−0.742, 0.502)	−1.96

EG2, experimental group with augmented reality and multimodal training; EG1, experimental group with multimodal training; PA, physical activity; MVPA, moderate-vigorous physical activity.

* Wilcoxon test *p*-value.

** Paired-samples *t*-test *p*-value.

*** *p* ≤ 0.05.

No significant differences were observed between groups for upper limb flexibility, cardiorespiratory fitness, dominant handgrip, or in the light, moderate, vigorous, moderate-to-vigorous physical activity (MVPA), and total physical activity levels.

## 4 Discussion

The first purpose of this study was to investigate the effects of a multimodal exercise program and a multimodal exercise program with AR in FF and PA. As the population ages, developing strategies that promote health and quality of life is essential. Nowadays, technology is used in the daily life of a large part of the general population and has become an important aspect of people's lives. Multimodal exercise benefits functional fitness and physical activity in older people, but few studies use technology in conjunction with exercise sessions. This study demonstrated that a multimodal program incorporating AR can lead to significant improvements in functional fitness among community-dwelling older adults. Our findings indicate notable increases in various aspects of functional fitness, and both intervention programs contributed to enhancements in specific functional skills, resulting in clinically significant effects.

When comparing the three groups, differences were found in upper and lower limb strength, lower limb flexibility, timed up and go, functional reach test, non-dominant hand grip, and balance. When we compared EG2 with CG, changes were observed in upper and lower limb strength, balance, and timed up and go, indicating that an AR intervention has multiple benefits in terms of functional fitness. When we compared EG1 with CG, we observed differences between the groups in the variables of functional reach test, lower limb flexibility, balance, and timed up and go. When comparing the experimental groups with the control group, significant differences were found in some variables. This can be attributed to the fact that the control group did not undergo any specific intervention, while the experimental groups participated in a 12-week exercise intervention. These findings align with previous studies that utilized a CG without an intervention, highlighting the positive effects of structured exercise programs on functional fitness in older adults (34, 35). In contrast, when comparing EG2 and EG1, EG1 participants showed better results in the FRT. EG2 showed improvements in non-dominant hand grip (HGNDH) compared to EG1. In EG1, participants showed a decrease in hand pressure strength (Δ% = −3%), whereas participants in EG2 increased it with exercise (Δ% = 9%). This could be because activities with AR require using both upper limbs to perform the activities successfully.

When comparing the pre- and post-training assessments within each group in the present study, we identified significant differences in functional fitness across all groups. In the control group (CG), participants exhibited declines in functional fitness variables, particularly in timed up and go (TUG) performance and upper limb flexibility. These findings align with other studies that indicate a decrease in functional fitness with advancing age.

When comparisons were made between the pre- and post-training moment within each group in the present study, we found some significant differences in functional fitness in all groups. In the CG, participants exhibited declines in functional fitness variables, particularly in timed up and go performance and upper limb flexibility. These findings align with other studies that indicate a decrease in functional fitness with advancing age (36–38). EG2 demonstrated improvements in upper and lower limb strength, lower limb flexibility, timed up and go, cardiorespiratory fitness, balance, and non-dominant hand grip strength. In contrast, EG1 showed enhancements in lower limb flexibility, timed up and go, balance, and cardiorespiratory fitness. Although there were differences within each group between the pre- and post-exercise assessments, the effect size was found to be greater in EG2. Clinically relevant effects were also more pronounced in EG2. However, it is important to note that the intervention with only multimodal training still showed clinically relevant effects in certain variables. According to our results, both interventions demonstrated clinically significant improvements across different areas. EG1 achieved better outcomes in balance, cardiorespiratory fitness, and lower limb flexibility, while EG2 excelled in strength and cardiorespiratory fitness. An earlier study by Park and Shin showed that an AR intervention improved the timed up and go of older women living in the community (23). However, it did not show improvements in the five times sit-to-stand and the 1-min sit-to-stand test, designed to measure lower limb function. According to the authors, this may be related to the fact that the exercise program developed did not consist of strength exercises. In this study, large muscle groups were used in the work of the group that used augmented reality. All PEPE activities required participants to perform movements with the lower and upper limbs simultaneously, and some loading was added during the sessions to increase the difficulty. In contrast to the results obtained in Park, we improved lower and upper limb strength. To our knowledge, this is one of the few studies that used an AR exercise platform that allows participants to perform exercises in the real world with the addition of virtual elements.

This study shows that although both experimental groups showed improvements in several variables EG2 produced more results and had several clinically relevant effect. The use of AR in the training sessions provided participants with constant stimulation and immediate feedback. Our training sessions used 4 PEPE activities that trained agility, cardiorespiratory fitness, upper and lower limb muscle strength, and motor skills. All activities required participants to perform movements with their legs (lateral displacements, stationary walking, or squats) and with their arms (rotation, extension and flexion, adduction, and abduction) while maintaining a constant movement. As the training sessions progressed, the exercises were adapted, and the load of the exercises increased. One of the advantages of using PEPE is the constant feedback provided to the participants during the execution of the exercises. A previous study by Gonçalves et al. (21), which observed the effects of using PEPE on physical activity levels, concluded that this platform proved to be an effective addition to exercise sessions for ageing. They demonstrated that AR training sessions could promote a higher percentage of time spent in moderate-vigorous physical activity than traditional training sessions. The fact that more moderate-vigorous physical activity occurs leads to greater significant gains among participants in this study (22, 39, 40).

Regarding physical activity, within each group, there were significant differences between baseline and end-line only at EG1 in sedentary behavior and sleep duration. However, participants had worse results, increasing sedentary behavior time (Δ% = −7%), and decreasing sleep time (Δ% = −9%). When we compare the 3 groups, differences are observed in the same 2 variables, with differences between EG2 and EG1. Although there were no significant differences in EG2, there was a trend toward an increase in sleep time (Δ% = 6%) and a decrease in sedentary time (Δ% = −5%). To our knowledge, no studies have examined the effects of an AR exercise program on sedentary behavior and sleep in community-dwelling older adults. However, previous studies have shown that after an exercise program, older people decrease the amount of time spent in sedentary behavior (41) behavior and improve their sleep quality (42). In the future, it will be essential to assess sleep quality and efficiency to understand what impact a program at AR might have on these variables and to develop strategies for participants to remain physically active outside of sessions.

The integration of multimodal training with AR presents significant potential for inclusion in public health strategies and interventions focused on enhancing functional fitness and reducing sedentary behavior among older adults. AR can counteract the adverse effects of a sedentary lifestyle by offering interactive and engaging exercise experiences. Public health initiatives could leverage AR by incorporating it into programs designed to reduce sedentary behavior in older populations, especially through creative implementations in senior centers and community spaces. The findings indicate that technology-enabled fitness programs can address multiple facets of functional fitness, making them a valuable element of active aging strategies. Policies that encourage the use of AR technologies in community-based exercise programs could lead to improvements in physical fitness and, consequently, the overall health and well-being of older adults. This approach has the potential to lower healthcare costs associated with physical decline and to support aging in place.

This study has several limitations. Firstly, monitoring participants’ heart rates throughout the exercise sessions would have been crucial for a more precise understanding of their physiological responses. Future research should consider incorporating heart rate monitors to better assess exercise intensity. Another limitation is that the sample was not randomly assigned; instead, group allocation was based on participants' availability. Additionally, the conclusion of the intervention coincided with the summer season, and high temperatures at the intervention site may have reduced participants’ motivation to engage in physical activity, potentially impacting the study's outcomes.

## 5 Conclusion

The results of this study indicate that participation in a multimodal exercise program that incorporates augmented reality (AR) leads to significant functional improvements in older adults. The multimodal exercise program enhanced with AR proved particularly effective, yielding broader and more substantial improvements in several functional fitness variables compared to a program that did not utilize this technology. Understanding the motivations of older adults in community settings to engage in physical exercise is crucial for enhancing the effectiveness of these programs. AR has shown promise as a tool for increasing engagement by allowing older adults to interact with virtual objects within their real-world environments, thereby making exercise more enjoyable and accessible. Integrating AR into exercise programs for community-dwelling older adults can result in a variety of health benefits, including improved physical function, heightened motivation, and potentially reduced sedentary behavior.

## Data Availability

The data supporting the conclusions of this article is not ready available due to participant confidentiality. Requests to access the data can be directed to the corresponding author.
